# Formation and Thermal Behaviors of Ternary Silicon Oxycarbides derived from Silsesquioxane Derivatives

**DOI:** 10.3390/ma12101721

**Published:** 2019-05-27

**Authors:** Yoshiaki Iwase, Teruaki Fuchigami, Yoji Horie, Yusuke Daiko, Sawao Honda, Yuji Iwamoto

**Affiliations:** 1 Applied Research Laboratory, General Center of Research and Development, Toagosei Co., Ltd., 8, Showa-cho, Minato-ku, Nagoya 455-0026, Japan; yoshiaki_iwase@mail.toagosei.co.jp (Y.I.); youji_horie@mail.toagosei.co.jp (Y.H.); 2 Department of Life Science and Applied Chemistry, Graduate School of Engineering, Nagoya Institute of Technology, Gokiso-cho, Showa-ku, Nagoya 466-8555, Japan; fuchigami.teruaki@nitech.ac.jp (T.F.); daiko.yusuke@nitech.ac.jp (Y.D.); honda@nitech.ac.jp (S.H.)

**Keywords:** silicon oxycarbide, amorphous state, thermal stability, crystallization, silsesquioxane, Polymer-Derived Ceramics (PDCs)

## Abstract

Silsesquioxane (SQ) derivatives possessing intramolecular H_2_C = CH- groups and Si-H groups were designed as precursors for ternary silicon oxycarbide (SiOC). By using R-Si(OMe)_3_, H-Si(OEt)_3_ and (H-Si(Me)_2_)_2_O as starting compounds, SQ derivatives of VH-SQ (R = vinyl) and St-H-SQ (R = stylyl) were successfully synthesized through the conventional sol-gel route. Simultaneous thermogravimetric and mass spectroscopic analyses up to 1000 °C revealed that in situ cross-linking via hydrosilylation and demethanation of VH-SQ suppressed the evolution of gaseous hydrocarbon species to afford amorphous SiOC having a composition close to the desired stoichiometric SiO_2(1−x)_C_x_ (x = ca. 0.3) with a high yield. The effect of carbon content on the phase separation and crystallization of the SQ-derived amorphous SiOC was studied by several spectroscopic analyses and TEM observation. The results were discussed aiming to develop a novel polymer-derived ceramics (PDCs) route for in situ formation of binary β-SiC-amorphous SiO_2_ nanocomposites with enhanced thermal and mechanical stability.

## 1. Introduction

**Figure 1:**
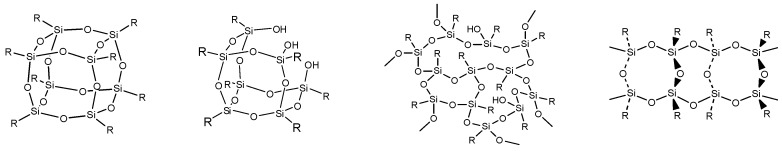
General structures of silsesquioxanes (SQs).

Recently, increasing attention has been directed to organic–inorganic hybrid materials as promising functional materials in diverse fields such as optics, electronics, housing and energy. Synergistic properties of hybrid materials can be achieved by harmonizing advantageous properties of an organic component, such as solubility, plasticity and hydrophobicity, with those of an inorganic component, namely, mechanical strength and thermal and chemical stability [1,2]. Silsesquioxane (SQ) derivatives (Figure 1) are typical organic–inorganic hybrid materials that have been widely known as commercial products, including hard coatings, protective coatings for spacecraft and gate insulating films, with each required property which tuned by modifying its composition at the molecular scale level [3,4,5,6].

Silsesquioxane derivatives have been also found as a useful polymer precursor for ternary silicon oxycarbides (SiOC) composed of an amorphous network of corner-connected SiO_x_C_4−x_ (x = 0–4) tetrahedral [7]. The chemical composition of polymer-derived SiOC materials can be tuned by incorporating appropriate organic substituents and controlling cross-linking degrees of polymer precursors prior to pyrolysis under inert atmosphere. Typically, carbon-rich silicon oxycarbides have been synthesized [7,8,9]. They exhibit an excellent thermal stability in terms of keeping amorphous states beyond 1000 °C [9]. At around 1200–1400 °C, amorphous SiOC begins to start phase separation and crystallization. One possible pathway is the formation of amorphous SiO_2_, β-SiC and amorphous/graphitic carbon without the evolution of any gaseous products (Equation (1)). Then, further crystallization of β-SiC proceeds via a decomposition reaction between SiO_2_ and carbon, according to Equation (2). Finally, the decomposition completes to afford a stoichiometric mixture of amorphous SiO_2_ and β-SiC (Equation (3)) [9,10,11,12].

Amorphous SiOC → SiO_2_(a) + xSiC(β) + yC(a/gra) (1)

SiO_2_(a) + 3C → SiC(β) + 2CO(g) ↑ (2)

SiO_2_(a) + xSiC(β) + yC(a/gra) → (1–y/3)SiO_2_(a) + (x + y/3)SiC(β) + 2/3CO (g) ↑ (3)

**Figure 2:**
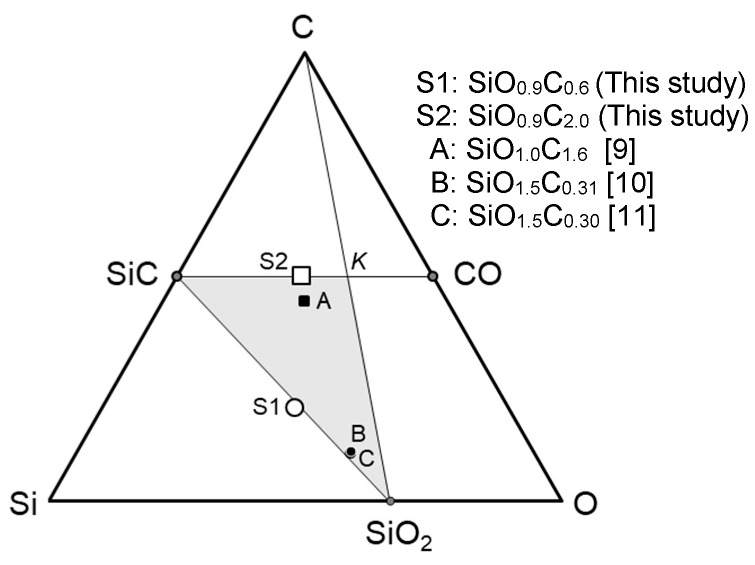
Chemical compositions of the polymer-derived amorphous SiOC reported in literature [9,10,11], and those of the S1 and S2 derived from SQs synthesized through the sol-gel route shown in Equation (4).

Saha and Raj proposed a practical method for predicting the maximum crystallization and weight loss for amorphous SiOC materials located within the SiC-K-SiO_2_ triangle shown in the Si-O-C composition diagram (Figure 2), and it was reported that the thermal behavior in terms of β-SiC crystallization and CO evolution up to 1350 °C of the polymer-derived amorphous SiO_1.0_C_1.6_, labeled as (A) in Figure 2, was well consistent with their prediction [9].

For improving the thermal and mechanical stability of SiC-amorphous SiO_2_ composites, it is attractive to synthesize amorphous SiOC with a stoichiometric composition (SiO_2(1−x)_C_x_) located on the tie line between SiC and SiO_2_ to afford a segregated carbon-free SiC-SiO_2_ composite (according to Equation (1) without excess carbon). Heat treatment at 1400 °C of amorphous SiO_1.5_C_0.31_ possessed a composition with relatively low carbon content and was close to the SiC-SiO_2_ tie line (labeled as (B) in Figure 2), resulting in the formation of binary β-SiC nanocrystallites finely dispersed amorphous SiO_2_ [10]. By hot-pressing at 1400 °C, amorphous SiO_1.5_C_0.30_ (labeled as (C) in Figure 2) was also converted to such a segregated carbon-free nanocomposite, which exhibited a relatively low coefficient of thermal expansion (ca. 3.2 × 10^−6^ K^−1^), low thermal conductivity (ca. 1.5 W·m^−1^·K^−1^) and high viscosity approximately two orders of magnitude higher than that of vitreous silica [11,12].

In this study, amorphous SiO_2(1−x)_C_x_ with a relatively high carbon content (labeled as S1 in Figure 2)—approximately two times higher compared with those of the previous (B) and (C) in Figure 2—was synthesized together with a reference material of carbon-rich SiOC (labeled as S2 in Figure 2 comparable with (A) in Figure 2 previously investigated by Saha and Raj [9].

For the synthesis of S1 and S2, silsesquioxane (SQ) derivatives which have intramolecular H_2_C = CH- groups and Si-H groups labeled as VH-SQ and St-H-SQ were designed respectively, and synthesized through the conventional sol-gel route (Equation (4)). The thermal decomposition of organic substituents, as well as the thermally induced in situ cross-linking during pyrolysis of the SQ derivatives, were characterized. Then, phase separation and crystallization of the SQ-derived amorphous SiOC were studied and discussed, with the intention of developing a novel polymer-derived ceramic (PDC) route for the in situ formation of binary β-SiC- amorphous SiO_2_ nanocomposites.

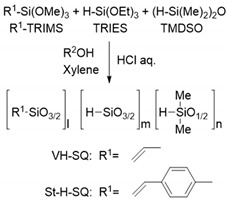
(4)

## 2. Experimental Section

### 2.1. Precursor Synthesis

The handling of all the reagents and products in this study was performed under inert atmosphere of pure nitrogen (N_2_). VH-SQ and St-H-SQ were synthesized through the sol-gel route (Equation (4)) according to the procedures described in the patents released from Toagosei Co., Ltd. [6]. A 500-mL four-necked flask equipped with a dropping funnel, a magnetic stirrer and a septum, was charged with vinyl-trimethoxysilane (V-TRIMS, 10.4 g, 70 mmol, TCI, Tokyo, Japan), triethoxysilane (TRIES, 34.5 g, 210 mmol, Toagosei Co. Ltd., Tokyo, Japan), tetramethyldisiloxane (TMDSO, 9.38 g, 70 mmol, TCI, Tokyo, Japan), 2-propanol (40 g, Wako Pure Chemical Industry Ltd., Osaka, Japan) and xylene (120 g, TCI, Tokyo, Japan). Through the funnel, the mixture of aqueous HCl (diluted to 1.28%, 16.5 g, Kishida Chemical Co. Ltd., Osaka, Japan) and 2-propanol (20 g) was added dropwise at 20 °C over 30 min. The mixture was allowed to stand overnight at around 25 °C. Then, the solvent was removed under vacuum below 60 °C to afford VH-SQ as a colorless paste (20.5 g, 96% yield). 

By using stylyl-trimethoxysilane (St-TRIMS, 31.4 g, 140 mmol, TCI, Tokyo, Japan) instead of V-TRIMS, St-H-SQ was synthesized under the same manner (colorless solid, 27.5 g, 92% yield).

### 2.2. Pyrolysis and Heat Treatment

The synthesized SQ was placed in an alumina boat and pyrolyzed in a quartz tube furnace under flowing nitrogen (N_2_, 200 mL/min) by heating from room temperature to 800 °C in 2 h, then maintaining for an additional 1 h and finally furnace-cooling down to room temperature to create a solid product. to afford a product as a solid.

The pyrolyzed sample was ground to a fine powder using mortar and pestle. The powdered sample was placed on a carbon crucible and heat-treated in a graphite resistance-heated furnace (Model High Multi 5000, Fujidempa Kogyo, Osaka, Japan) under vacuum from room temperature to 500 °C. Then, Ar gas was introduced into the furnace at 500 °C and the temperature was increased to 1400, 1600 or 1800 °C and held for an additional 1 h. The heating rate was 10 °C/min. The Ar pressure applied in this heat treatment from 500 to 1800 °C was 98 kPa. After the heat treatment, the sample was cooled down to room temperature in the furnace. 

### 2.3. Characterizations

The attenuated total reflection–infra red (ATR-IR) spectra were recorded on as-synthesized SQs with a diamond prism under an incidence angle of 45° (Model Spectrum 100, Perkin Elmer, Waltham, MA, USA). 

^29^Si nuclear magnetic resonance (NMR) spectra were acquired at 79.5 MHz (Model ECA-400, JEOL, Tokyo, Japan). The spectra for as-synthesized SQs were recorded in CDCl_3_ solution at room temperature, and the chemical shifts were referenced to tetramethylsilane (0 ppm). For pyrolyzed and heat-treated samples, solid-state ^29^Si magic angle spinning (MAS) NMR spectra were acquired at a rotation frequency of 15 kHz, and the chemical shifts were quoted relative to the signals of 3-(trimethylsilyl) propionic acid sodium salt (2 ppm).

Thermal behaviors up to 1000 °C were studied by simultaneous thermogravimetric (TG) and mass spectroscopic (MS) analyses (Model TG/DTA 6300, Hitachi High Technologies Ltd., Tokyo, Japan/Model JMS-Q1050GC, JEOL, Tokyo, Japan). The measurements were performed under flowing helium (100 mL/min) with a heating rate of 20 °C/min. Conventional thermogravimetric and differential thermal analyses (TG/DTA) analyses were also performed in N_2_ to study their exothermal behaviors.

Elemental analyses were performed on the pyrolyzed or heat-treated samples for oxygen, nitrogen and hydrogen (inert-gas fusion method, Model EMGA-930, HORIBA Ltd., Kyoto, Japan), and carbon (non-dispersive infrared method, Model CS844, LECO Co., St Joseph, MI, USA). The silicon content in the samples was calculated as the difference of the sum of the measured C, N, O and H content to 100 wt%. 

X-ray diffraction (XRD) measurements were performed on pyrolyzed or heat-treated samples (Model X’pert Pro α1, Philips Ltd., Amsterdam, Nederland).

Raman spectra were recorded on the pyrolyzed or heat-treated samples (Model NRS-3300, Jasco, Tokyo, Japan) using a 532-nm solid laser.

Crystallization behavior of the polymer-derived amorphous SiOC materials was observed by using a transmission electron microscope (TEM, Model JEM-2100F, JEOL, Tokyo, Japan, operating at 200 kV). Analytical investigations were carried out by electron energy loss spectroscopy (EELS, Model Enfina, Gatan, Inc., Pleasanton, CA, USA). 

## 3. Results and Discussion

### 3.1. SQ Preceramic Polymers

**Figure 3:**
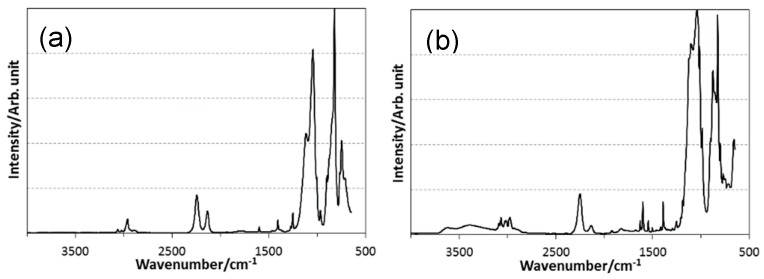
ATR-IR spectra for (**a**) VH-SQ and (**b**) St-H-SQ.

**Figure 4:**
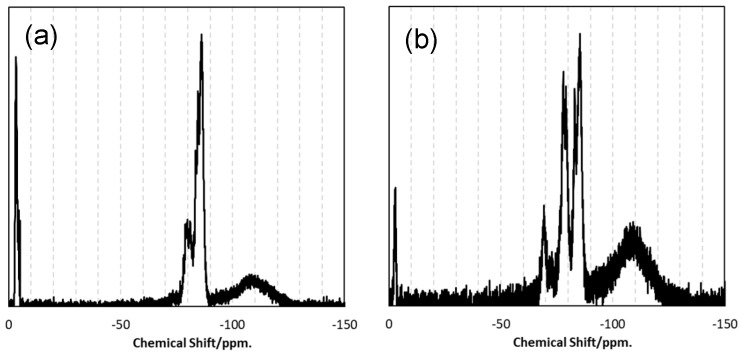
^29^Si NMR spectra for (**a**) VH-SQ and (**b**) St-H-SQ.

The chemical structures of VH-SQ and St-H-SQ were identified by the ATR-IR and ^29^Si NMR spectra shown in Figure 3 and Figure 4, respectively.

ATR-IR spectra for as-synthesized SQs presented characteristic absorption bands at 2136 and 2252 cm^−1^ assigned to Si-H derived from TMDSO and TRIES, respectively, with those at 1392 and 1602 cm^−1^ attributed to the C = C bond (Figure 3a,b). The intensity of the broad peak at around 3376 cm^−1^ indicated in St-H-SQ (Figure 3b) was relatively higher than that in VH-SQ (Figure 3a). This could be due to the contribution of C-H stretching in phenyl moiety of the styryl group. On the other hand, the broad band at around 3300 to 3700 cm^−1^ was assigned to the Si-OH group (Figure 3b).

The ^29^Si NMR spectrum for VH-SQ presented characteristic peaks attributed to the units composing the ternary SiOC organic–inorganic hybrid network at −3.3, −79.5 to −81.1, and −86.0 ppm, assigned to M1 (O-Si(CH_3_)_2_H), T3^Vi^ ((≡SiO)_3_Si-Vi) and T3^H^ ((≡SiO)_3_Si-H), respectively [13]. Note that another very wide and weak peak centered at around −110 ppm was the background (i.e., Q4 (Si (OSi≡)_4_) unit derived from the glass sample tube (Figure 4a). In addition to these peaks, St-H-SQ presented a broad peak centered at −68.8 ppm assigned to the T2^St^ ((≡SiO)_2_(MeO)_2_Si-St) unit [13] (Figure 4b). However, compared with VH-SQ, the relative peak intensity of the M1 unit at −2.9 ppm was apparently lower. This feature suggests that the number of the terminate M1 units of St-H-SQ was smaller than that of VH-SQ, which could lead to the detection of (≡SiO)_3_Si-OH by the ATR-IR spectroscopic analysis (Figure 4b).

These results revealed that VH-SQ and St-H-SQ had possessing a common main architecture with different compositions that was successfully synthesized as designed. Since the residual OR and Si-OH groups were easily eliminated during pyrolysis up to 800 °C, the SQ derivatives synthesized in this study were converted to inorganic SiOC without further purification

### 3.2. Pyrolytic Behavior

**Figure 5:**
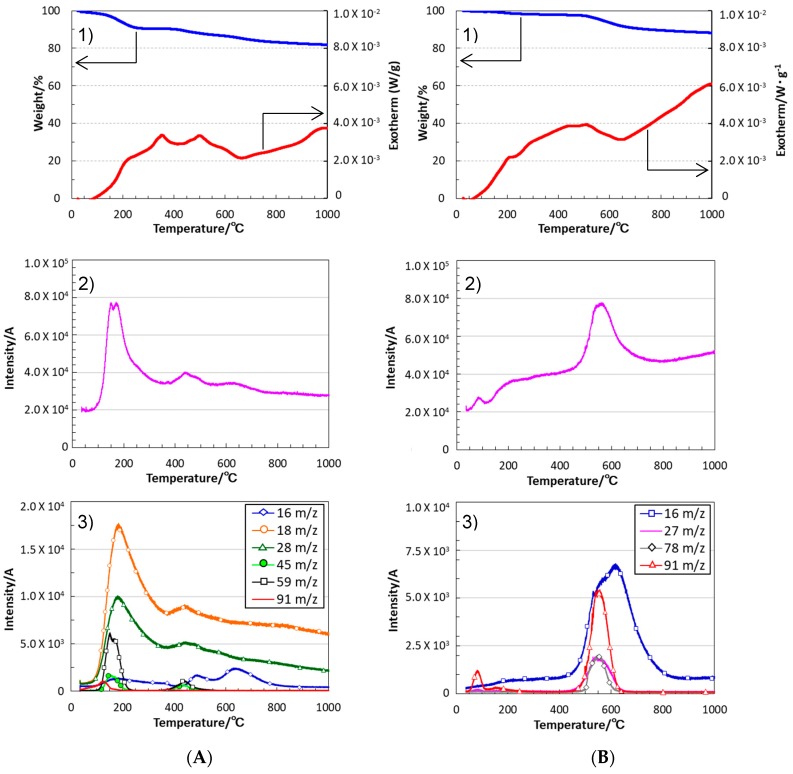
Thermal behavior of (**A**) VH-SQ and (**B**) St-H-SQ under a He flow. (**1**) TG/DTA curves, (**2**) total ion current chromatogram (TICC) detected for the gaseous species formed in situ, and (**3**) continuous in situ monitoring of the evolved gaseous species by mass spectroscopy.

**Figure 6:**
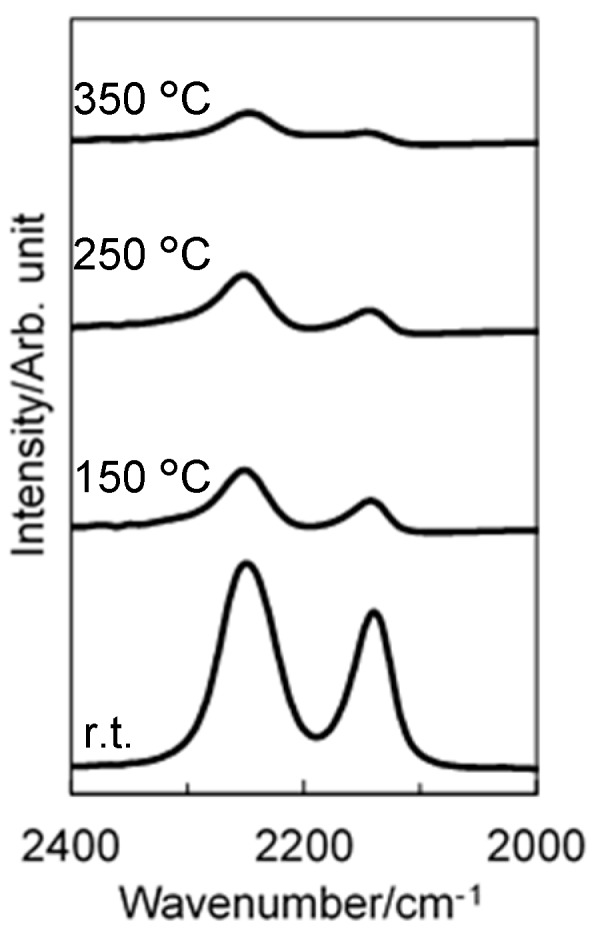
ATR-IR spectra for as-synthesized VH-SQ (room temperature) and those after heat treatment at 150 to 350 °C in N_2_.

The SQ derivatives showed high ceramic yields at 1000 °C, and 82% and 88% were achieved for VH-SQ and St-H-SQ, respectively. Each of them exhibited a DTA curve with a broad exothermic peak at around 200 to 600 °C (Figure 5A1,B1). Figure 6 shows the typical results obtained for VH-SQ. According to the ATR-IR spectroscopic analysis, the band intensities of 2136 and 2252 cm^−1^ which assigned to Si-H groups of SQ derivatives remarkably decreased in this temperature range. Thus the broad exothermic peak could be attributed mainly to hydrosilylation (Figure 5A1,B1). 

VH-SQ presented two weight-loss regions, 10% up to 300 °C and 8% at around 400–800 °C (Figure 5A1). The gas evolution was mainly detected at the first weight-loss region (Figure 5A2), and several gaseous species were simultaneously detected (Figure 5A3). The m/z ratio at 18 could be assigned to H_2_O^+^ formed by the dehydration condensation between the Si-OH groups. Another element with the m/z ratio at 28 was assigned to C_2_H_4_^+^ as a fragment of the organic group formed by the hydrosilylation. On the other hand, the m/z ratio at 91 was a fragment of xylene (i.e., residual solvent). Those at 59 and 45 could also be fragments that originated from another residual solvent, 2-propanol. However, it should be noted that the m/z ratio at 59, detected at around 400 °C, could be assigned another possible element, C_2_H_7_Si^+^ (HMe_2_Si^+^), formed in situ by the thermal decomposition of the TMDSO unit. The m/z ratio at 16, detected at around 400–800 °C, was assigned to CH_4_^+^ evolved by the demethanation between the Si-CH_3_ groups. Accordingly, the first weight loss up to 300 °C was due to the evaporation of the residual solvents and H_2_O, while the cross-linking through the hydrosilylation and demethanation efficiently proceeded up to 800 °C, which led to the high ceramic yield at 1000 °C.

In the case of St-H-SQ, the main gas evolution shifted to higher temperatures ranging from 450 to 650 °C, and the cross-linking via demethanation was found to proceed at 450 to 800 °C by detecting CH_4_^+^ (m/z = 16). Other gas species with m/z ratios at 91 (CH_2_-C_6_H_5_^+^) and 78 (C_6_H_6_^+^) that evolved above 450 °C were fragments of the organic group formed by hydrosilylation, while that at 27 (C_2_H_3_^+^) could have originated from the remaining vinyl group (Figure 5B2,3).

Chemical compositions of the 800 °C-pyrolyzed products are listed in Table 1. As reference data, theoretical compositions of as-synthesized SQ derivatives are also listed in this table. Besides high ceramic yield, the in situ cross-linking of VH-SQ led to suppressing the thermal decomposition of organic groups, which lead to the formation of inorganic SiOC with the desired carbon content, labeled as S1. Moreover, by replacing the vinyl group with the styryl group in the SQ, the amount of carbon in the SiOC successfully increased to afford S2.

### 3.3. Crystallization Behaviors of SQ-Derived Amorphous SiOC

**Figure 7:**
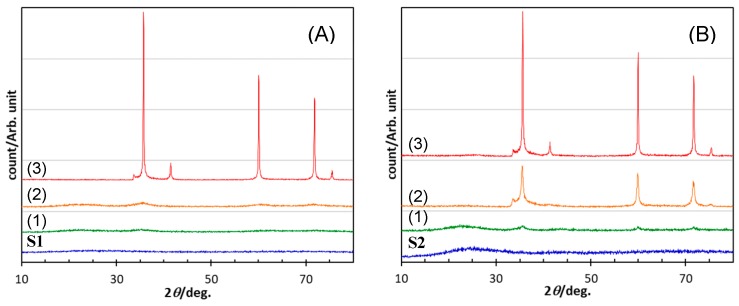
XRD patterns of S1 and S2 synthesized by pyrolysis at 800 °C, and those after heat treatment at (**1**) 1400, (**2**) 1600 and (**3**) 1800 °C. Polymeric precursors: (**A**) VH-SQ and (**B**) St-H-SQ.

**Figure 8:**
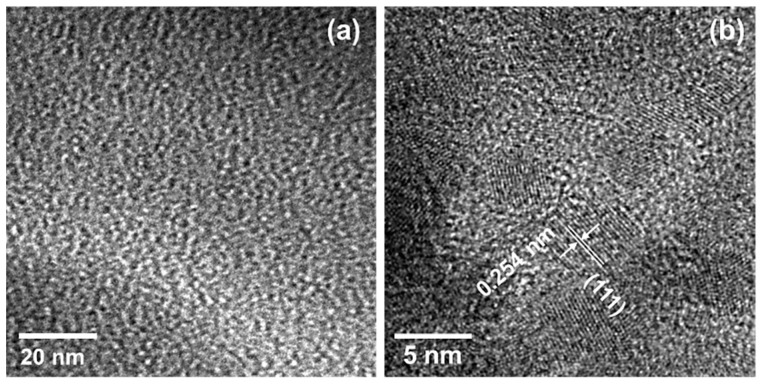
TEM images of the VH-SQ-derived S1 after heat treatment at (**a**) 1400 and (**b**) 1600 °C.

As shown in Figure 7, S1 and S2 were X-ray amorphous. During heat treatment, they began to show diffraction peaks that increased in intensity with increasing temperature. Finally, the 1800 °C heat-treated samples exhibited distinct diffraction peaks at 2θ (degrees) of 35.8, 41.5, 60.0, 71.9 and 75.8, indexed to (111), (200), (211), (311) and (222) of β-SiC (JCPDS Card No. 29–1129), respectively. The weaker peak at 33.7 could be attributed to the stacking fault of β-SiC. The β-SiC crystallization onset temperatures of S1 and S2 were observed above and below 1400 °C, respectively. Then, TEM analysis was performed on the heat-treated S1 samples. As shown in Figure 8a, the 1400 °C-heated S1 exhibited a futureless structure typical for amorphous compounds. Then, after the 1600 °C heat treatment, some crystallites several nanometers in size were observed within the amorphous matrix. The inter planer spacing observed for the nanocrystallite formed in situ was measured to be 0.254 nm, which corresponded to the (111) plane of β-SiC (JCPDS card no. 29–1129) (Figure 8b). Accordingly, the S1 kept the amorphous state up to 1400 °C, and the β-SiC crystallization onset temperature was found to be higher than that of S2.

**Figure 9:**
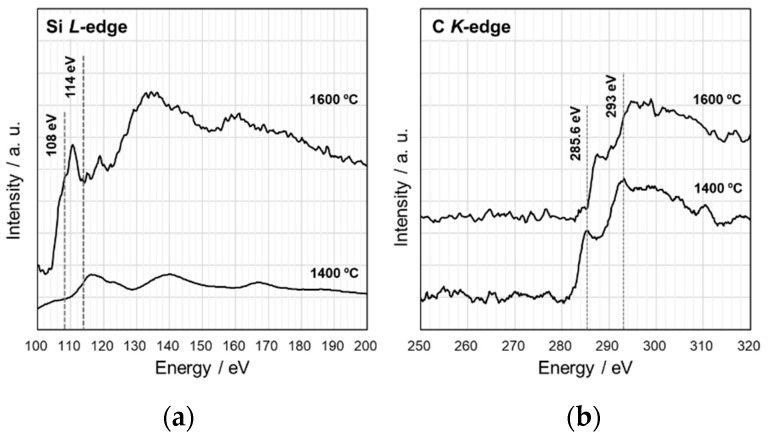
EELS spectra for the SQ-derived S1 after heat treatment at 1400 and 1600 °C. (**a**) Si L-edge and (**b**) C K-edge.

**Figure 10:**
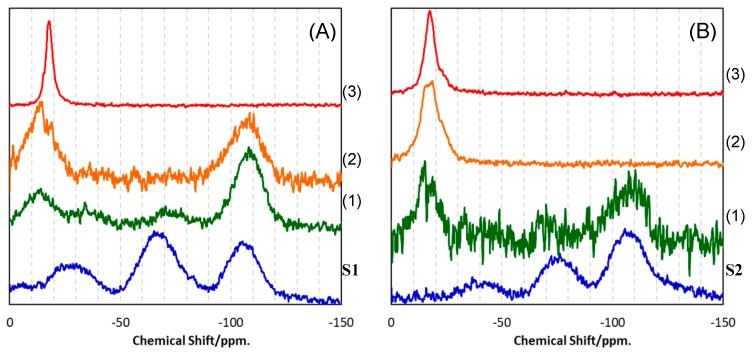
^29^Si MAS NMR spectra for SQ-derived S1 and S2 synthesized by pyrolysis at 800 °C, and those after subsequent heat treatment at (**1**) 1400, (**2**) 1600 and (**3**) 1800 °C. Polymeric precursors: (**A**) VH-SQ and (**B**) St-H-SQ.

To study the β-SiC crystallization behavior in more details, EELS analysis was performed on the heat-treated S1 (Figure 9). After heat treatment at 1400 °C, the Si L_2,3_ edge exhibited a featureless line, then at 1600 °C the spectrum presented characteristic peaks at about 108 and 114 eV, related to 2p-p* and 2p-d* transitions in SiO_2_ [14,15]. It is noteworthy that the distinct peak at around 110 eV suggested that some Si bonded to both O and C partly remained [15]. On the other hand, after heat treatment at 1400 °C, the C K-edge showed the spectrum typical for amorphous carbon (i.e., two peaks due to the 1s-π* (285.6 eV) and 1s–σ* (293 eV) transitions, respectively [16]). Then, at 1600 °C, the two peaks remarkably decreased in intensity. These results suggest that phase separation to amorphous/graphitic carbon and amorphous SiO_2_ dominantly proceeded prior to β-SiC crystallization. This behavior is supported by the results obtained by Raman, as well as by ^29^Si MAS NMR spectroscopic analyses. The Raman spectra for the heat-treated S1 samples (Figure 10A) indicated the formation of amorphous/graphitic carbon at 1400 and 1600 °C by detecting two distinct peaks at 1347.5 and 1596.5 cm^−1^, which were attributed to the D-band (for disordered graphite) and G-band (for the sp^2^ graphite network), respectively [17,18]. Two minor broad peaks centered at 2682.5 and 2933.5 cm^−1^ were attributed to the disordered carbon, assigned to the two-dimensional (2D) band and the D + G band, respectively [17]. At 1800 °C, the peaks attributed to the amorphous/graphitic carbon almost disappeared and new peaks appeared at 797.5 and 972 cm^−1^ (weak), which were typical for β-SiC and corresponded to the transverse (TO) and longitudinal (LO) optical modes, respectively [19]. 

The ^29^Si MAS NMR spectrum for as-synthesized S1 presented three broad peaks centered at about −30, −70 and −105 ppm and assigned to SiO_2_C_2_, SiO_3_C and SiO_4_ units, respectively [20,21]. At 1400 °C, the broad peak assigned to the SiO_4_ units remained, while the peaks due to the mixed tetrahedral SiO_2−x_C_x_ (x = 1, 2) units disappeared, and a new broad peak appeared at about −EELS15 ppm attributed to the SiC_4_ unit, which was typical for disordered SiC [22]. At 1600 °C, another new peak appeared at about 18 ppm, which indicated the formation of SiC with improved crystallinity as a β phase [22]. At 1800 °C, the spectrum presented one distinct peak attributed to β-SiC (Figure 10A3). 

**Figure 11:**
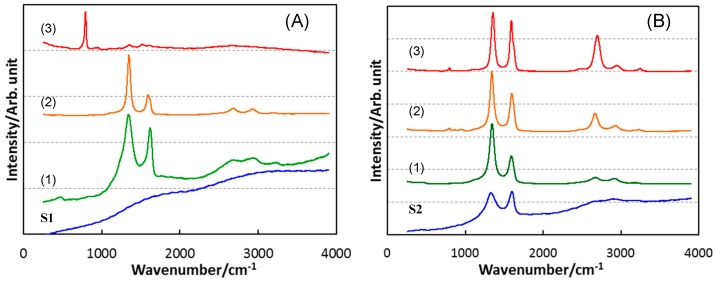
Raman spectra for SQ-derived S1 and S2 synthesized by pyrolysis at 800 °C, and those after subsequent heat treatment at (**1**) 1400, (**2**) 1600 and (**3**) 1800 °C. Polymeric precursors: (**A**) VH-SQ and (**B**) St-H-SQ.

Raman, as well as the ^29^Si MAS NMR spectroscopic analyses for heat-treated S2 samples, revealed similar phase separation and subsequent β-SiC crystallization (Figure 10B and Figure 11B). However, the amorphous/graphitic carbon segregation had already started during pyrolysis up to 800 °C, and remained at all temperatures from 1400 to 1800 °C.

The S1 synthesized by pyrolysis at 800 °C of VH-SQ was amorphous SiOC single phase, while S2 was a binary composite of amorphous SiO_2_ and amorphous/graphitic carbon. The phase separation and crystallization pathway up to 1400–1600 °C observed in the present study was different from that previously suggested for polymer-derived amorphous SiOC materials as expressed by Equations (1)–(3) [9,10,11,12]. Regardless of carbon content in terms of stoichiometric S1 (SiO_2(1−x)_C_x_) or S2 (SiO_2(1−x)_C_x_ + yC), the dominant pathway could be expressed by the following reactions: phase separation to amorphous/graphitic carbon and amorphous SiO_2_ (Equation (5)), then subsequent β-SiC crystallization via carbothermal reduction of SiO_2_ (Equation (2)) to afford binary β-SiC and amorphous SiO_2_ (Equation (6)).

xSiO_2_(a) + yC(a/gra) (5)

(x − y/3)SiO_2_(a) + ySiC(β) + 2y/3CO(g) ↑ (6)

2SiO_2_(a) + SiC(β) → 3SiO(g) ↑ + CO(g) ↑ (7)

(y − (x − y/3)/2) SiC(β) + 3(x − y/3)/2SiO(g) ↑ + (x − y/3)/2CO(g) ↑(8)

The VH-SQ-derived S1 was found to keep an amorphous state up to 1400 °C. This enhanced thermal stability could be achieved by the phase separation (Equation (5)) associated with the local structure rearrangement from SiO_2−x_C_x_ (x = 1, 2) to carbon-free SiO_4_ tetrahedral prior to the formation of SiC_4_ units, which are essential for the nucleation and crystallization of β-SiC. 

On the other hand, in the case of the St-H-SQ-derived S2 having excess carbon, the initial phase separation (Equation (5)) had already started during pyrolysis up to 800 °C, which could lead to β-SiC crystallization below 1400 °C. 

**Figure 12:**
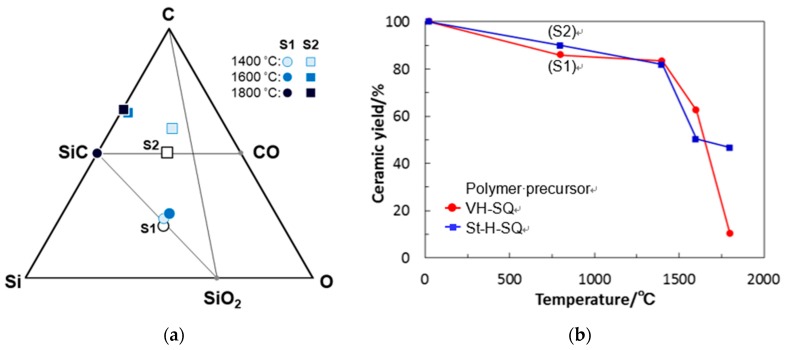
Thermal behaviors of VH-SQ-derived S1 and St-H-SQ-derived S2 during heat treatment up to 1800 °C in Ar. (**a**) Chemical composition changes and (**b**) ceramic yields.

Further heat treatment above 1400 °C of the SQ-derived SiOC materials led to apparent weight loss and a remarkable decrease in oxygen content (Table 1, Figure 12a,b). This was mainly the result of a thermodynamically favorable reaction between SiO_2_ and SiC, which yielded gaseous SiO and CO (Equation (7)) [23]. The amorphous SiO_2_ in the S1 could be spent out to afford single-phase β-SiC by heat treatment up to 1800 °C, while this reaction in the S2 almost completed at 1600 °C. As a result, the final 1800 °C heat-treated product was β-SiC with free carbon.

## 4. Summary

In this study, silsesquioxane (SQ) derivatives possessing intramolecular H_2_C = CH- groups and Si-H groups were designed and synthesized as precursors for stoichiometric amorphous SiO_2(1−x)_C_x_ with relatively high carbon content (x > 0.15). Chemical structures and pyrolysis behaviors of the SQ derivatives, as well as thermal behaviors up to 1800 °C in Ar of the SQ-derived amorphous SiOC materials, were investigated. The results can be summarized as follows:

(1) ATR-IR and ^29^Si-NMR spectroscopic analyses revealed that the SQ derivatives VH-SQ (R = vinyl) and St-H-SQ (R = stylyl) were successfully synthesized through the conventional sol-gel route using R-Si(OMe)_3_, H-Si(OEt)_3_ and (H-Si(Me)_2_)_2_O as starting compounds.
(2) The simultaneous TG-MS analyses showed that, under an inert atmosphere, thermally induced cross-linking via hydrosilylation and demethanation was efficiently achieved. The resulting ceramic yields after heating VH-SQ and St-H-SQ to 1000 °C were 82% and 88%, respectively.
(3) Besides of the high ceramic yield, the in-situ cross-linking of VH-SQ led to suppressing the evolution of gaseous species that originated from hydrocarbon substituents up to 800 °C, leading to the formation of amorphous SiOC (labeled as S1) with a desired composition close to the stoichiometric SiO_2(1−x)_C_x_ (x = ca. 0.3).
(4) By replacing the vinyl group with the styryl group, the carbon content in the amorphous SiOC successfully increased to afford SiO_0.9_C_2.0_H_0.8_ with excess carbon (labeled as S2).
(5) Heat treatment up to 1800 °C in Ar of the amorphous SiOC materials revealed that, regardless of carbon content (in terms of stoichiometric S1 or S2 having excess carbon), the dominant pathway for β-SiC crystallization could be expressed by the following reactions: phase separation to amorphous/graphitic carbon and amorphous SiO_2_, followed by β-SiC crystallization via carbothermal reduction of SiO_2_.
(6) The VH-SQ-derived S1 was found to hold an amorphous state up to 1400 °C. This enhanced thermal stability could be achieved by the phase separation associated with the local structure rearrangement from SiO_2−x_C_x_ (x = 1, 2) to carbon-free SiO_4_ tetrahedral prior to the formation of SiC_4_ units essential for the nucleation and crystallization of β-SiC.
(7) Further heat treatment above 1400 °C of the SQ-derived SiOC materials led to large weight loss and a remarkable decrease in oxygen content, which could be due to the thermodynamically favorable reaction of SiO_2_ with SiC to yield gaseous SiO and CO.
(8) The VH-SQ was found to be a useful precursor for synthesizing amorphous SiOC with enhanced thermal stability. However, for the formation of β-SiC–SiO_2_ nanocomposite having a higher fraction of β-SiC nanocrystallites, it is important to facilitate the nucleation of SiC prior to phase separation to amorphous/graphitic carbon and amorphous SiO_2_. Currently, further study of the relationship between the chemical state of carbon and SiC nucleation within the amorphous SiOC, as well as heat treatment conditions to facilitate SiC nucleation, are in progress.

## Figures and Tables

**Table 1:** Chemical composition of pyrolyzed and heat-treated samples.

Sample	Composition [wt%]				SiOC Composition Empirical Ratio SiO_2(1−x)_C_x_ + yC_free_ or ySi_free_
	Si	O	C	H	
As-synthesized VH-SQ	45.9	34.1	15.7	4.26	SiO_1.3_C_0.8_H_2.6_ (SiO_1.3_C_0.35_ + 0.45C_free_)
**S1:** 800 °C-pyrolyzed VH-SQ	57.4	28.9	12.8	0.94	SiO_0.9_C_0.5_H_0.5_ (SiO_0.9_C_0.53_ + 0.05Si_free_)
**S1:** 1400 °C-heat treated	55.4	29.5	15.1	0.08	SiO_0.9_C_0.6_H_0.0_ SiO_0.9_C_0.55_ + 0.05C_free_
**S1:** 1600 °C-heat treated	55.4	30.6	15.1	0.03	SiO_1.0_C_0.7_H_0.0_ SiO_0.9_C_0.50_ + 0.2C_free_
**S1:** 1800 °C-heat treated	55.4	0.24	29.7	0.02	SiC_1.0_O_0.0_H_0.0_ SiC
As-synthesized St-H-SQ	27.8	23.0	45.2	4.06	SiO_1.5_C_3.8_H_4.1_ (SiO_1.5_C_0.25_ + 3.55C_free_)
**S2:** 800 °C-pyrolyzed St-H-SQ	41.5	22.4	34.9	1.15	SiO_0.9_C_2.0_H_0.8_ (SiO_0.9_C_0.55_ + 1.45C_free_)
**S2:** 1400 °C-heat treated	33.4	21.2	45.4	0.13	SiO_1.1_C_3.2_H_0.1_ SiO_1.1_C_0.45_ + 2.75C_free_
**S2:** 1600 °C-heat treated	51.1	2.28	46.6	0.23	SiO_0.1_C_2.1_H_0.1_ SiO_1.0_C_0.95_ + 1.15C_free_
**S2:** 1800 °C-heat treated	52.5	0.28	47.2	0.04	SiO_0.0_C_2.1_H_0.0_ SiC + 1.10C_free_
